# Barriers and facilitators to implementing bubble CPAP to improve neonatal health in sub-Saharan Africa: a systematic review

**DOI:** 10.1186/s40985-020-00124-7

**Published:** 2020-04-28

**Authors:** Mai-Lei Woo Kinshella, Celia R. Walker, Tamanda Hiwa, Marianne Vidler, Alinane Linda Nyondo-Mipando, Queen Dube, David M. Goldfarb, Kondwani Kawaza

**Affiliations:** 1 Department of Obstetrics and Gynaecology, BC Children’s and Women’s Hospital and University of British Columbia, Vancouver, Canada; 2 Department of Pathology and Laboratory Medicine, BC Children’s and Women’s Hospital and University of British Columbia, Vancouver, Canada; 3 Department of Pediatrics and Child Health, College of Medicine, University of Malawi, Blantyre, Malawi; 4 School of Public Health and Family Medicine, Department of Health Systems and Policy, College of Medicine, University of Malawi, Blantyre, Malawi; 5 Department of Paediatrics, Queen Elizabeth Central Hospital, Blantyre, Malawi

**Keywords:** Bubble continuous positive airway pressure (CPAP), Sub-Saharan Africa, Neonates, Implementation, Low-resource settings

## Abstract

**Background:**

Bubble continuous positive airway pressure (CPAP) has been shown to be effective in supporting breathing in newborns with respiratory distress. The factors that influence implementation in resource-constrained settings remain unclear. The objective of this review is to evaluate the barriers and facilitators of CPAP implementation for newborn care at sub-Saharan African health facilities and how different facility levels and types of bubble CPAP systems may impact utilization.

**Methods:**

A systematic search (database inception to July 2019) was performed on MEDLINE Ovid, EMBASE, CINAHL, The Cochrane Central Register of Controlled Trials (CENTRAL, The Cochrane Library), the WHO Regional Database for Africa, African Index Medicus (AIM), African Journals Online, grey literature and the references of relevant articles. Studies that met the inclusion criteria (primary research, bubble CPAP implementation with neonates ≤ 28 days old at a health facility in sub-Saharan Africa) were included in the review and assessed with National Heart, Lung, and Blood Institute of the National Institutes of Health (NIH) quality assessment tools. The review protocol was published to PROSPERO (CRD42018116082).

**Results:**

Seventeen studies were included in the review. Reliable availability of equipment, effectively informing and engaging caregivers and staffing shortages were frequently mentioned barriers to the implementation of bubble CPAP. Understaffed neonatal units and high turnover of nurses and doctors compromised effective training. Provider-to-provider clinical mentorship models as well as affordability and cost-effectiveness of innovative bubble CPAP systems were identified as frequently mentioned facilitators of implementation.

**Conclusions:**

With a strong recommendation by the World Health Organization for its use with premature infants with respiratory distress, it is important to understand the barriers and facilitators that can inform the implementation of bubble CPAP. More research is needed into health system factors that can support or impede the use of this potentially promising intervention.

## Introduction

Globally, there have been significant declines in infant mortality but rates of neonatal mortality are declining at a slower pace than among older infants and children [1]. Although the third Sustainable Development Goal (SDG-3) aims to reduce under-five mortality rates to fewer than 25 per 1000 live births and neonatal mortality rates to fewer than 12 per 1000 by the year 2030, a recent study found that if the slow trend in neonatal mortality reduction continues, only two out of the 31 sub-Saharan African countries are predicted to achieve SDG-3 targets [2]. Among newborns in sub-Saharan Africa, one in every 36 neonates die within the first month, a staggering inequality compared to one in 333 in high-income countries (HICs) [3]. Preterm birth complications are a leading cause of neonatal death [1] and a review found that nine of the 11 countries globally with estimated preterm birth rates of 15% or more were in sub-Saharan Africa [4]. With an estimated average pre-term birth rate at 12.3% across the sub-Saharan Africa region (12.3%), there is a need to effectively address accompanying complications in order to reduce the burden of neonatal deaths [1, 4]. Preterm newborns often have underdeveloped respiratory systems, with more than 50% of infants born before 31 weeks gestation developing respiratory distress syndrome (RDS) [5].

Newborns with RDS can be managed effectively with breathing support, such as mechanical ventilation or continuous positive airway pressure (CPAP), as well as surfactant replacement therapy [6]. CPAP is strongly recommended by the World Health Organization (WHO) for the treatment of preterm newborns with RDS [7]. CPAP is a simple, non-invasive form of respiratory support requiring less advanced technical expertise than mechanical ventilation, an invasive procedure involving endotracheal intubation or tracheostomy tube insertion [8–11]. Although CPAP is widely recommended for managing respiratory distress and has been utilized in high-income countries (HICs) for decades, hospitals in resource-limited settings still experience challenges in its implementation. Conventional CPAP machines, while less costly than mechanical ventilation, are still not economically sustainable in most low- and middle-income countries (LMICs). Bubble CPAP provides a potential solution, as a safe and cost-effective way for delivering CPAP in LMICs [8]. Bubble CPAP safely regulates air pressure by submerging the end of the expiration tubing into water, with the depth of the tube in the water determining the pressure in the system [8–12]. This maintains a volume of air in the lungs (functional residual lung capacity) to support the newborn’s spontaneous breathing [8–12]. A systematic review demonstrated that when bubble CPAP is utilized effectively, it can reduce the need for mechanical ventilation by 30–50% with no increase in mortality [8].

Although there is evidence suggesting that bubble CPAP is an efficacious, safe, cost-effective device to support neonatal breathing, there is still a lack of knowledge regarding factors influencing implementation in limited-resource settings [13]. Consequently, the primary objective of this systematic review is to evaluate known barriers and facilitators to the implementation, utilization and sustainability of bubble CPAP for neonates at health facilities in sub-Saharan Africa. Secondary objectives of the review explore the varying types of devices and the different hospital settings where bubble CPAP are used. The secondary objectives are firstly, to scope and document the different types of bubble CPAP and facility levels implementing bubble CPAP in sub-Saharan Africa and secondly, to evaluate barriers and facilitators of bubble CPAP for neonates at health facilities in sub-Saharan Africa by health facility and device type. The third secondary objective is to understand the efficacy and safety of bubble CPAP for neonates at health facilities in sub-Saharan Africa by health facility and device type. Based on the strong recommendation by the WHO, it is important to understand the barriers and facilitators that can inform effective bubble CPAP implementation in resource-constrained settings such as in many sub-Saharan hospitals, where bubble CPAP therapy is a critical need.

## Methodology

Searches were undertaken on MEDLINE Ovid, EMBASE, CINAHL, The Cochrane Central Register of Controlled Trials (CENTRAL, The Cochrane Library), the WHO Regional Database for Africa, African Index Medicus (AIM) and the African Journals Online database to July 2019 by the primary (MWK) reviewer, with no limits applied to the year of publication. Based on the PICOS research framework (Table 1), search terms broadly included infants, neonates, respiratory distress syndrome (RDS), sub-Saharan Africa and bubble CPAP (see Additional file 1). Results were then manually screened for implementation, utilization and health facility to prevent missing relevant studies in the original search that did not include these keywords. Grey literature, including programme reports and dissertations, was searched on Google and Google Scholar and conference proceedings authors were contacted directly by the primary researcher. Reference lists of all primary studies were reviewed for additional references.

**Table 1:** PICOS research framework

**P**opulation	Neonates ≤ 28 days
**I**ntervention	Bubble CPAP
**C**ontext	Secondary or tertiary health facilities in sub-Saharan Africa
**C**omparisons	Non-bubble CPAP, other respiratory support interventions, no respiratory support interventions, N/A
**O**utcome	Enablers and barriers of bubble CPAP, survival to discharge rates
**S**tudy	All study designs

Titles and/or abstracts of studies were independently screened by the primary and secondary reviewers (MWK and CRW) according to the eligibility criteria (Table 2). We included studies that self-identified as a bubble CPAP device though recognize that a recent study has shown that a low-cost standalone system, Pumani, does not fully follow the mechanisms of a bubble CPAP device [14]. However, because this study focuses on implementation factors and Pumani has been updated in response to the study by Falk and colleagues, we have elected to include it in our review as the broader picture of health system barriers and facilitators remain similar. Discussions of efficacy and safety will present separate data for Pumani device. Barriers were defined as implementation factors that hindered CPAP use within local contexts while facilitators were implementation factors that supported CPAP use as self-reported by studies. Reviewers compared their results to reach consensus and ties were resolved by a third reviewer (THM). Full text of these studies was then independently reviewed by the primary and secondary reviewers, with the third reviewer (THM) providing an independent assessment in any disagreements regarding eligibility until consensus was reached. Details about the study method, sample size, country, facility type, bubble CPAP type, outcomes, complications, barriers, and facilitators were extracted into a data extraction sheet. The ROBINS-I tool was initially planned for quality assessment but its focus on non-randomized studies was not suited to the diversity of studies found in the search. The focus of the ROBINS-1 tool is on intervention studies [15], which was too narrow of a scope to accommodate the variety of observational studies that highlight implementation aspects. The study quality assessment tools of the National Heart, Lung, and Blood Institute of the National Institutes of Health (NIH) for quality assessment of case series, observational cohort and cross-sectional studies, before-after studies with no control group and controlled intervention studies were used to assess the quality of included studies [16, 17]. The review protocol was published to PROSPERO (CRD42018116082) [18].

**Table 2:** Eligibility criteria

Inclusion criteria	Exclusion criteria
• Discussed implementation and/or utilization of self-identified bubble CPAP systems with neonates (≤ 28 days old) • At a health facility in sub-Saharan Africa • Randomized controlled trials, quasi-experimental studies, observational and exploratory studies, case studies, economic evaluations, programme reports and clinical charting	• Neonatal data not separated from older infants older than 28 days, older children and/or adults • Studies that do not specify bubble CPAP from other forms of CPAP or neonatal respiratory support systems • Studies without primary data collection on using bubble CPAP in a health facility • Study protocols, literature reviews, conference proceedings, letters to the editor, opinion papers, editorials and abstracts • Not in English

## Results

**Fig. 1:**
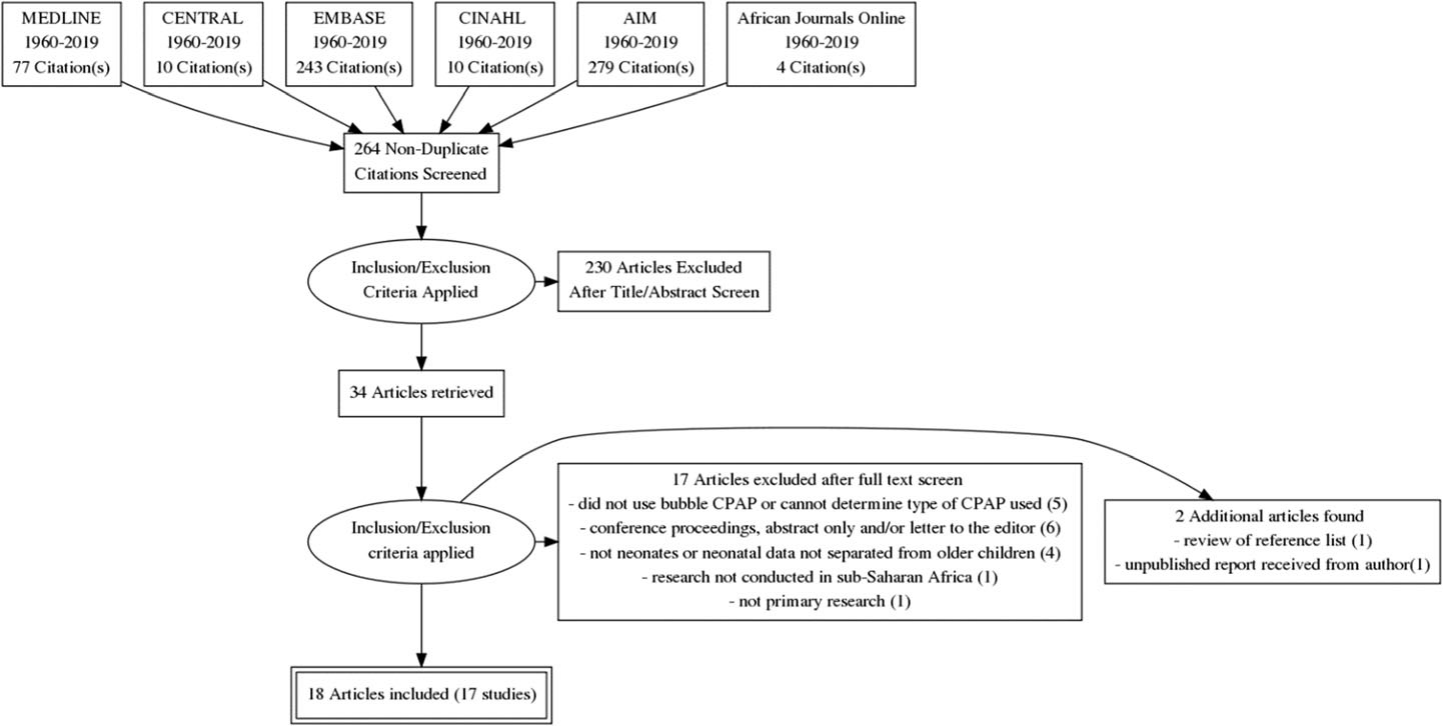
PRISMA flow diagram

Seventeen studies that discussed using bubble CPAP at a health facility in sub-Saharan Africa with neonates were found in the search (Fig. 1, Table 3). This included six case studies or series of neonates who received bubble CPAP [19, 20, 22, 23, 29, 34], four observational cohort or cross-sectional studies [26, 30, 31, 33], three uncontrolled before-and-after studies [12, 25, 32] and four case-control intervention studies that compared novel use of bubble CPAP systems or implementation components with local standards of existing care [5, 21, 24, 28]. All four case-control intervention studies were quasi-experimental and there were no randomized controlled trials.

**Table 3:** Studies included in the review

Reference	Research design	Sample	Location	Facility level	Device used	Quality assessment
Abdulkadir et al. [19]	Descriptive case study	Male newborn (1 h old) with idiopathic respiratory distress syndrome	Ahmadu Bello University Teaching Hospital (ABUTH), Nigeria	Tertiary	Bubble CPAP system not described	Poor quality
Abdulkadir et al. [20]	Descriptive case series of neonates who received nasal bubble CPAP	20 spontaneously breathing newborns with respiratory distress over 1-year period from 1 June 2012 to 31 May 2013	Ahmadu Bello University Teaching Hospital (ABUTH), Nigeria	Tertiary	Improvised water bottle system	Fair quality
Amadi et al. [21]	Quasi-experimental study comparing politeCPAP outcomes with standard care (improvised bubble CPAP system)	57 neonates with RDS who met eligibility from three hospitals, dates unknown	Multiple locations, Nigeria	Tertiary	Low-cost standalone system—politeCPAP	Poor quality
Audu et al. [22]	Descriptive case series of neonates who received nasal bubble CPAP	48 babies admitted into newborn unit with respiratory distress over a 6-month period, dates unknown	National Hospital Abuja, Nigeria	Tertiary	Improvised water bottle system	Fair quality
Brown et al. [23]	Descriptive case study	A full-term neonate with respiratory distress caused by congenital pneumonia	Queen Elizabeth Central Hospital, Malawi	Tertiary	Low-cost standalone system—Pumani	Poor quality
Crehan et al. [24]	Quasi-experimental study comparing nurses’ assessments with new decision-aid with a paediatrician’s assessment	57 neonates who received joint assessments by nurses and paediatrician, 27 April to 15 June 2015	Zomba District Hospital, Malawi	Secondary	Low-cost standalone system—Pumani	Poor quality
Fulton and Lavalette [25]	Pre and post study with follow-up 6 months after interventions introduced	58 neonates in October 2012 and 55 neonates in February 2013	Felege Hiwot Referral Hospital, Ethiopia	Tertiary	Improvised water bottle system	Poor quality
Gondwe et al. [26]	Observational phenomenological study with in-depth semi-structured interviews	12 caregivers of infants in Chatinkha nursery (0–28 days) and paediatric nursery (0–6 months) that improved on bCPAP January to February 2015	Queen Elizabeth Central Hospital, Malawi	Tertiary	Low-cost standalone system—Pumani	Poor quality
Kawaza et al. [5], Chen et al. [27]	Quasi-experimental study with allocation to CPAP based on availability of equipment [5] with an economic evaluation [28]	87 neonates (62 bCPAP and 25 nasal oxygen) over a 10 month period from January 2012 to October 2012	Queen Elizabeth Central Hospital, Malawi	Tertiary	Low-cost standalone system—Pumani	Fair quality
McAdams et al. [29]	Descriptive case series of neonates who received nasal bubble CPAP	21 neonates starting < 3 days of age in NICU from January to June 2012	Kiwoko Hospital, Uganda	Secondary (rural referral hospital)	Improvised water bottle system	Fair quality
Myhre et al. [12]	Pre and post introduction of bCPAP retrospective chart review	All preterm infants diagnosed with RDS consisting of 46 infants enrolled from 1 November 2007 to 30 April 2009 before and 72 infants enrolled from 1 November 2009 to 30 April 2011 after introduction of bCPAP	AIC Kijabe Hospital, Kenya	Secondary (rural referral hospital)	Improvised water bottle system	Fair quality
Nabwera et al. [30]	Observational cross-sectional survey with a structured assessment tool and qualitative key informant interviews and focus group discussions	39 neonates who received bubble CPAP between March and May 2017; 19 (of 23) tertiary level hospitals in Kenya surveyed	Multiple locations, Kenya	Tertiary level hospitals	Majority (93%) used commercial bubble CPAP systems	Fair quality
Nahimana et al. [31]	Observational retrospective chart review of all newborns admitted to neonatal units in three rural hospitals	43 infants initiated on bubble CPAP admitted between 1 February to 31 October 2013 (136 preterm and very low birthweight admitted overall)	Butaro, Kirehe and Rwinkwavu District Hospitals, Rwanda	Secondary (rural district hospitals)	Bubble CPAP system not described	Fair quality
Ntigurirwa et al. [32]	Pre and post clinical audits with follow-up 18 months after interventions introduced	365 infants in the first 18 months of introduction between February 2012 and January 2014	Two university hospitals and two district hospitals, Rwanda	Tertiary and secondary	Commercial bubble CPAP system—Fisher Paykel	Fair quality
Okonkwo and Okolo [33]	Observational cross-sectional survey administered to attendees during the 2015 Paediatric Association of Nigeria Conference (PANCONF)	237 questionnaires returned by doctors and nurses	54 health facilities from six geopolitical regions of Nigeria	Mostly public (87%) tertiary hospitals (76%)	Improvised water bottle system vs commercial bubble CPAP system—Fisher Paykel	Poor quality
Olayo et al. [28]	Quasi-experimental study that compared knowledge and skill of first and second-generation health professionals trained	37 (16 nurses, 21 physicians, medical/clinical officers) first-generation trained July 2014 to August 2015 and 40 (19 nurses, 21 physicians, medical/clinical officers) second generation	Multiple locations, Kenya	Tertiary	Commercial bubble CPAP system—DeVilbiss IntelliPAP	Poor quality
van den Heuvel et al. [34]	Descriptive case series of neonates who received nasal bubble CPAP	11 neonates during a 7-week introduction period from 11 March to 27 April 2008	Queen Elizabeth Central Hospital, Malawi	Tertiary	Improvised water bottle system	Fair quality

Of the 17 studies included in this review, six (35%) used improvised water bottle systems, five (29%) used low-cost standalone systems, two (12%) used commercial bubble CPAP system and four (24%) did not describe the system used or covered a combination of different types of devices. Four out of the five low-cost standalone systems used the Pumani bubble CPAP device, which was developed in Malawi [5, 23, 24, 26], and one study used politeCPAP, which was recently developed in Nigeria [21]. DeVilbiss IntelliPAP [28] and Fisher Paykel [32] bubble CPAP systems were the two commercial versions used. Most studies were conducted in tertiary level hospitals (11 studies; 65%), with four (24%) conducted in secondary level rural referral or district hospitals and two (12%) included both tertiary and district hospitals. Studies were largely clustered in East African countries, Malawi and Nigeria. All the studies were published 2011 or afterwards.

### Device characteristics

The device characteristics that promoted the use of bubble CPAP included the fact that it was simple to use compared with mechanical ventilation and that it was affordable and low maintenance [19, 20, 22]. Pricing ranged from US$2 for a low-cost, improvised water bottle system made from locally available materials, US$350 for a low-cost standalone unit developed in Malawi, to US$2000 for a standalone unit including twin air compressors, temperature-controlled gas delivery and integrated pulse oximeter developed in Nigeria [5, 19–22, 27, 33].

Two studies, both from tertiary facilities in Nigeria, raised the concern that efficacy of bubble CPAP may be limited to mild to moderate respiratory distress and less complex cases [19, 20]. These studies found that the majority of the newborns that did not respond to CPAP had a high Downes’ score, which marks respiratory distress severity, and had a very (less than 1500 g) or extremely (less than 1000 g) low birthweight or other severe conditions [19, 20]. Extremely low-birthweight newborns are at heightened risk for neonatal hypothermia and one study demonstrated that a bubble CPAP system with temperature-controlled gas delivery had the potential to reduce the risk of neonatal hypothermia, especially with this population [21].

### Training and staffing

An important barrier for the implementation of bubble CPAP was around understaffing of neonatal units. As discussed in five studies, a lack of adequate staffing may limit the capacity for care, especially if existing workloads were already substantial [24, 25, 29, 32, 34]. An early study of bubble CPAP in Malawi found that there were only two nurses available for an average of 30 neonates [34]. Understaffing was associated with limited availability for training, which was compounded with other challenges such as high turnover of nurses and doctors necessitating repeated trainings of new staff [25, 28, 30–32]. Consequently, studies noted gaps in the training that was available. A study in Nigeria found that 44% of respondents were untrained in the use of bubble CPAP even though the same respondents reported that CPAP was used in 72% of their facilities [33].

Additionally, four studies described challenges around motivation and leadership [25, 30–32]. Researchers from a study conducted in Ethiopia noted the challenge of poor motivation as nurses did not appear to be interested in practising what they had learned in trainings [25]. Lack of motivation was linked to poor accountability, understaffing and frequent rotations by medical and nursing leadership [25, 32]. Additionally, one study found that local nurses did not understand English well, which presented a communication barrier with doctors who conducted medical meetings and ward rounds in English [25]. Good leadership by hospital management who perceived neonatal care as a priority was identified as an important facilitating factor for bubble CPAP implementation in 19 tertiary level facilities across Kenya [30].

Four studies highlighted the importance of regular and interactive training and that it could serve as a facilitator to implementation [24, 25, 28, 31]. The effective training used a wide range of methods including comprehensive presentations on respiratory physiology, indications to initiate bubble CPAP, contraindications and monitoring complemented by instructional videos, the use of case examples, simulation scenarios with mannequins and real-time supervision and utilization of bubble CPAP on an infant [24, 25, 28, 31]. Provider-to-provider clinical mentorship models supported training efforts, especially when clinical mentors were trained in how to train others to use bubble CPAP [28, 30, 31]. Experience sharing trips to effective neonatal units and intermittent refresher trainings were helpful in supporting knowledge and skill development; this built motivation and morale as health providers regarded it as an investment in their professional capacities [25, 31]. One study recommended investing by training the nurses who across profession generally remain on the ward compared to doctors who may move around the hospital or between hospitals [25]. Another study recommended resources be allocated to introducing long-term clinicians to reinforce training [32]. To build momentum and motivation, two studies used a combination of external consultants with local clinicians to provide training [31, 34].

### Initiation, monitoring and weaning

Four studies discussed barriers to commencement of CPAP [20, 23, 31, 34]. Challenges around initiation highlighted that gaps in the correct identification of early/mild signs of respiratory distress [31] and the reluctance of nurses to initiate CPAP due to short staffing and/or desire to consult with a doctor first [34] were associated with a delay in initiating bubble CPAP, which was ultimately associated with less optimal outcomes [5]. Three studies further highlighted that regular monitoring is required to prevent and manage complications, such as nasal prong-related complications and “CPAP belly syndrome” [5, 20, 23]. Additionally, two studies discussed the challenges around weaning, including knowing when to wean, especially when human resources were limited [29], and the need to monitor closely after weaning to ensure the neonate is not desaturating [19].

Decision aids were described as potential facilitators to bubble CPAP use, including a clinical algorithm to aid in deciding to initiate [24] and using a respiratory severity score to monitor respiratory distress [29]. No potential facilitators for the weaning process were presented.

### Caregivers

Three studies, including two from Malawi and one from Kenya, discussed barriers to implementation around engaging caregivers [23, 26, 30]. Brown et al. found that parents in Malawi may be reluctant to allow infants to receive oxygen therapy because the need for oxygen was considered to cause poor outcomes [23]. A qualitative study researching caregiver experiences at a tertiary hospital in Malawi found that there were gaps in consenting parents before starting bubble CPAP, particularly since there was limited visiting hours to the neonatal intensive care unit (NICU) and caregivers often found their newborn already commenced on bubble CPAP [26]. Information about bubble CPAP was poorly provided to all caregivers, including the mother and close family members [26]. The caregivers’ experiences also shared concerns on how bubble CPAP may complicate mother-infant interactions [26]. It was noted that mothers were afraid to hold their infants because they were worried that they would not be able to see their child’s face and that the device would interrupt skin-to-skin contact [26]. A study from Kenya highlighted that peer support from the caregivers of newborns that had survived after being put on bubble CPAP was a particularly powerful opportunity to educate parents and guardians who are unsure about the system [30].

### Supplies and equipment

Three studies, all from Nigeria, discussed using appropriate and locally available equipment as facilitators for bubble CPAP implementation [19, 20, 22]. Appropriate equipment focused on nasal prongs and the importance of snug-fitting and soft nasal prongs to prevent nasal damage [19, 20].

Five studies, however, also identified how a reliable supply and availability of equipment was an implementation challenge [5, 23, 30, 32, 34]. Ancillary equipment, such as oxygen concentrators, was an issue; even though the low-cost standalone bubble CPAP model was developed for low-resource settings and was low maintenance, the oxygen concentrators associated with the device were not [5]. In one study, 40% of oxygen concentrators failed due to line voltage spikes [5]. Cost of disposable nasal prongs was also identified as a barrier in resource-constrained settings as was the availability of CPAP machines [5, 23, 34]. A Malawian study by Kawaza et al. noted that 31% (*n* = 12) of the neonates died when the CPAP system was occupied and the newborn did not receive CPAP [5]. Studies also highlighted diversity on the health landscape as different CPAP machines caused challenges in training, setup and maintenance [32]. A recent study from Kenya found that most bubble CPAP systems across the 19 tertiary level facilities evaluated were commercial versions donated by international partners. These devices encountered problems with maintenance as donor projects completed and support was withdrawn [30]. Additionally, inadequate infrastructure was associated with lower staff morale and motivation to use bubble CPAP [30] (Table 4).

**Table 4:** Implementation factors

Topic	Facilitators	Barriers
Device	• Simple to use, affordable and low maintenance for low-resource settings. • A temperature-controlled gas circuit may reduce the risk of hypothermia especially in extremely low-birthweight babies.	• Efficacy may be limited to mild to moderate respiratory distress and less effective with severe cases.
Training and staffing	• Regular and interactive training with intermittent refresher trainings. • Clinical mentorship with training on how to train others to use bubble CPAP. • Investing in nurses dedicated to the nursery. • Clinicians that stay longer term in the nursery. • Combination of external consultant with local clinicians as trainers. • Health facility management that prioritized neonatal care.	• Understaffed neonatal units limit the capacity for care. • Staffing shortages exacerbated by healthcare provider strikes in some locations. • High turnover of nurses and doctors necessitated repeated training of new staff. • Lack of motivation and accountability. • Gaps in training as many nurses and doctors are untrained in bubble CPAP. • Communication barriers between doctors and nurses.
Initiation	• Decision-making aided by clinical algorithm that is clearly posted by the machine.	• Gaps in correct identification of early and mild signs of distress. • Reluctance of nurses to initiate while short-staffed at night and without consulting a clinician. • Overtightening the chin strap can lead to facial swelling.
Monitoring	• Appropriate and regular monitoring. • Monitoring with pulse oximetry. • Monitoring respiratory distress with respiratory severity score.	• Complications such as CPAP belly syndrome and mucosal drying require regular monitoring and actions to prevent.
Weaning	None discussed.	• Knowing when to wean, especially when resources are limited. • A need to monitor closely after weaning to ensure the infant is not desaturating.
Caregivers	• Peer support from caregivers with positive experiences with bubble CPAP use on their own newborns.	• Local beliefs that the oxygen led to poor outcomes. • Poorly providing information to caregivers and gaps in consenting parents before starting bubble CPAP. • Bubble CPAP may complicate mother-infant interaction as mothers were afraid to hold babies, unable to see their infant’s faces and interrupted skin-to-skin contact.
Supplies and equipment	• Appropriate snug-fitting nasal prongs. • Soft nasal prongs. • Use of locally available materials.	• Cost of disposable nasal prongs. • Oxygen concentrators not always available. • CPAP machines not always available. • Different machines cause challenges in training, set up and maintenance. • Poor equipment maintenance once donors withdraw support.

### Barriers and facilitators of bubble CPAP by health facility and device type

Across the multiple settings and device types, understaffed neonatal units, high turnover of staff and low staff motivation and morale to use bubble CPAP were common barriers. Common facilitators were clinical mentorship and regular, interactive training. Decision aids to support the initiation and monitoring respiratory distress were highlighted as facilitators in secondary level facilities [24, 29]. Affordability was highlighted as a facilitator in both improvised water bottle systems and low-cost standalone models [5, 20–22, 27]. Studies focusing on improvised water bottle systems highlighted the benefit of being able to use locally available materials to manufacture the bubble CPAP device, yet a perceived barrier remains because most improvised water bottle systems and standalone models do not heat or humidify the air being provided to the newborn [5, 21, 23]. Additionally, low-cost standalone models highlighted the benefit of low maintenance, while inadequate maintenance was cited as a barrier especially for commercial models [5, 21, 30].

### Efficacy and safety of bubble CPAP by health facility and device type

Nine of the 17 studies (53%) presented novel data on survival to discharge rates. Survival to discharge rates with bubble CPAP varied widely between studies, ranging from 49 to 85% in tertiary hospitals [20, 22, 30, 34] and 42 to 85% in rural district level referral hospitals [12, 29, 31]. Additionally, survival to discharge rates for the Pumani device which was noted in two studies ranged from 56 to 71% [5, 24] and for the improvised water bottle system, the survival to discharge rate ranged from 52 to 85% over five studies [12, 20, 22, 29, 34]. One study that did not describe the bubble CPAP system they used reported a 42% survival rate and another study that surveyed multiple hospitals where commercial models were the most frequently utilized device reported a 49% survival rate [30]. Mechanical ventilation was not available on site in eight of the nine studies and only one of these eight had the option of referring to a higher-level facility with mechanical ventilation [31]. A survey in Kenya found that mechanical ventilation was only available in 37% tertiary level hospitals across the nation [30].

Across the various forms of bubble CPAP systems, there were no major complications reported. Studies highlighted that this may be in part due to a lack of necessary resources to assess for these complications such as routine x-rays, cranial ultrasounds or autopsies to ascertain the cause of death [5, 23, 30]. Nasal and facial irritation was the most commonly reported minor complication across different bubble CPAP systems and facility levels. For example, nasal irritation was reported in 14% of cases in a secondary level facility with an improvised water bottle system [29], 13% of neonates initially started on bubble CPAP in a tertiary level facility with a low-cost standalone model (Pumani) [5] and 13% of infants in a multi-sited study with both tertiary and secondary level facilities with a commercial model [32]. Nasal irritation was not well defined but some studies described it as slight soreness, abrasions, swelling, redness and mucosal erosion [29, 34].

## Discussion

The purpose of the review was to evaluate the barriers and facilitators to implementing bubble CPAP for newborn care at sub-Sahara African health facilities. Reliable availability of equipment, difficulties engaging and informing caregivers and staffing shortages were frequently mentioned barriers to the implementation of bubble CPAP. Understaffed neonatal units and high turnover of nurses and doctors limited capacity for care and was associated with gaps in training, which subsequently impacted initiation, monitoring and weaning. Provider-to-provider clinical mentorship models were identified as frequently mentioned facilitators of bubble CPAP implementation within the context of staffing shortages and high staff turnovers as they helped to address challenges in the aim to formally train and retain trained staff in neonatal units. Affordability and cost-effectiveness were also highlighted as important facilitators, especially for low-cost standalone units and improvised models. A cost-effectiveness study on the low-cost standalone unit from Malawi found an average cost per patient on bubble CPAP was US$29.29 compared to US$57.78 per patient on nasal oxygen [27]. Mechanical ventilation costs in LMICs was not available, though a study in the USA found that costs for a preterm infant ≤ 32 weeks gestation on mechanical ventilation in the USA was US$51,000–209,000 [35].

A previous review revealed that bubble CPAP may reduce the need for mechanical ventilation in LMICs [8]. This is of great importance in resource-constrained contexts where mechanical ventilation is not often available due to high costs, maintenance demands and the need for highly trained staff. However, to use mechanical ventilation as a comparator to evaluate bubble CPAP excludes the most resource-constrained contexts where mechanical ventilation is unavailable. Such criteria would exclude almost all of the studies in the present review, for example. The tertiary hospitals in sub-Saharan Africa covered by the studies in this review frequently lacked the resources for mechanical ventilation, which suggests that differences between LMICs may be just as important as those between HICs and LMICs. A focus on low-resource settings is particularly important as bubble CPAP is being scaled-up to secondary level rural district hospitals in sub-Saharan Africa where contexts may be even more resource-constrained.

Secondary objectives of this review investigated how different facility levels and types of bubble CPAP systems may impact utilization, expanding on previous reviews on the efficacy of CPAP in LMICs where multiple types of nasal CPAP systems and health facility levels were grouped together [8, 9]. Few major complications were reported regardless of the system type and facility setting and survival to discharge rates appeared to differ more between studies than between the device used and the setting. The studies in this review, however, revealed contextual differences that could influence implementation and sustainability. Though many studies were conducted in tertiary level facilities, two of the four studies completed in secondary level facilities highlighted how decision-making aids were feasible and helpful to support nurses’ decision to initiate an infant on bubble CPAP and to monitor the neonates’ level of respiratory distress. The discussion around decision-making aids was absent in studies conducted in tertiary level facilities perhaps in part because the tertiary level facilities had full-time paediatric specialists on staff to make major decisions whereas the nurseries in secondary level facilities were often nurse-led [31].

Sub-Saharan Africa is unique in the global CPAP landscape for the development of novel, low-cost standalone bubble CPAP systems in an attempt to reduce cost and bridge the gap between conventional commercial models and locally improvised systems. Though less expensive than mechanical ventilation, conventional commercial bubble CPAP systems cost between US$6000 and 18,000, which means that they continue to be unaffordable for resource-constrained hospitals in sub-Saharan Africa [5, 21, 23]. Additionally, most commercial systems available in these settings were donated without the means to maintain them sustainably [30]. Both studies in the review using conventional commercial systems involved external partners that brought in foreign consultants to initially train and set up the programme [28, 32]. Maintenance and programme sustainability for the commercial systems were key barriers after the donors withdrew support and the presence of different types of bubble CPAP system made it challenging for staff to be adequately trained for all the different devices and have all of the components to set up and expertise in equipment maintenance for the various systems [30, 32]. With concerns around equipment maintenance and sustainability for the commercial bubble CPAP system, both low-cost standalone models highlighted the affordability and low maintenance of their systems as valuable facilitators to their implementation in sub-Saharan Africa [5, 21].

While innovative, low-cost, standalone models sought to present solutions to barriers of affordability and maintenance, studies also revealed the basic necessity for a supportive health system environment. Understaffed neonatal units, gaps in training and shortages of ancillary supplies, such as disposable nasal prongs and oxygen concentrators, continued to provide significant challenges. In this, the situation with bubble CPAP is not unique. The need for adequate supplies, financing, trained health workers and service delivery coherence within and between health facilities has also been highlighted as health system barriers in the scale-up of other maternal and child health interventions across sub-Saharan Africa [36–38]. These barriers hinder the sustainability of health interventions after implementation, particularly in sub-Saharan Africa where there is a disproportionate share of the global disease burden and weak health systems sensitive to the fluctuations of donor-funding [37]. For the implementation of bubble CPAP and other neonatal care innovations, there is a need to consider health systems and understand the challenges a low-resource setting presents in providing care for a neonate including the processes involved and other factors that may impact the newborn’s health. A retrospective analysis of the non-randomized controlled trial in Malawi, for example, found that for survival outcomes, bubble CPAP was not sufficient as a single intervention if a preterm neonate was hypothermic in addition to respiratory distress [39]. The persistently high mortality rates reported in the neonatal bubble CPAP studies in this review underlines the importance of health system strengthening and the human and material resources required to improve care outcomes for small and sick newborns. Even though the study population was different, the study by McCollum et al. illustrates this point further [40]. In a large trial involving 644 older infants and children up to 6 years of age, McCollum et al. found no reduction in hospital mortality with bubble CPAP use (Fisher & Paykel commercial device) in a rural Malawian hospital that did not have daily physician supervision [40]. For effective implementation of bubble CPAP, comprehensive quality care is needed in addition to appropriate technologies.

One limitation of this review is the exclusion of non-English texts, which may have excluded some studies. A strength of this review includes the use of multiple reviewers, the searches on multiple databases and grey literature and use of quality assessments. Similar to previous systematic reviews on CPAP and bubble CPAP across LMICs, the present review did not find any randomized controlled trials [8, 9]. However, considering the strong recommendation for its use with preterm newborns in respiratory distress by the WHO [7] and neonatal experts in the field [41], it is unethical to withhold treatment in order to conduct an RCT comparing bubble CPAP and nasal oxygen therapy. There are grounds, however, to suggest that other RCTs would be beneficial in developing a robust evidence-base for the facilitators, such as an RCT assessing survival outcomes from bubble CPAP use in addition to provided nursing utilization guidelines compared to bubble CPAP with no utilization guidelines.

The quality assessment revealed that many studies had considerable risk of biases (see Additional file 2) but unlike the previous systematic reviews published on bubble CPAP [8, 9], the primary purpose of this review was not to evaluate efficacy. Some of the implementation challenges around staffing shortages, training gaps and missing data may introduce bias into studies; however, from an implementation science perspective, they importantly revealed health system barriers and facilitators. Research that focuses on barriers and facilitators of implementation and utilization as the primary objective is needed, especially to understand the views of health professionals that interact with bubble CPAP provisioning and oversight.

## Conclusion

To accelerate progress towards the reduction of neonatal mortality in sub-Saharan Africa, improvements in neonatal care is needed, especially for premature newborns. There has been increasing interest in the utilization of bubble CPAP in resource-constrained health facilities in sub-Saharan Africa to improve outcomes for neonates with respiratory distress. However, the implementation of bubble CPAP is not without its challenges. While bubble CPAP may be seemingly streamlined and uncomplicated compared to mechanical ventilation, even simple and innovative technologies require supportive health systems for effective implementation and utilization. Future research into health system barriers and facilitators of bubble CPAP implementation is paramount for practical purposes, especially in low-resource and rural contexts.

## Supplementary information

**Additional file 1:.** Search terms.

**Additional file 2:.** Quality assessment tables.

**Additional file 3:.** Primary objective.

**Additional file 4:.** Secondary objectives.

## Data Availability

See Additional file 1 for search terms. See Additional file 2 for quality assessment tables. See Additional file 3 for primary objective tables. See Additional file 4 for secondary objective tables. Any additional data will be available upon request to the corresponding author.
