# Supplementary material for: Barriers and facilitators to implementing bubble CPAP to improve neonatal health in sub-Saharan Africa: a systematic review
Source: Public Health Rev. 2020 Apr 28;41:6. doi: 10.1186/s40985-020-00124-7 (PMC7189679; doi:10.1186/s40985-020-00124-7)
Supplement: Supplementary file 1 — Additional file 1:. Search terms. [file 40985_2020_124_MOESM1_ESM.docx]

**Additional file 1: Search terms**

| **Searches** | **Search terms** |
| --- | --- |
| Population | infant, newborn/ or infant, low birth weight/ or infant, small for gestational age/ or infant, very low birth weight/ or infant, postmature/ or infant, premature/  (infant or newborn or neonate or neonatal or premature or low birth weight or VLBW or LBW or infant* or neonat*).mp. not animals/  Respiratory Distress Syndrome, Newborn/ or (respiratory distress syndrome or RDS).mp |
| Setting | "Africa South of the Sahara"/  (sub-sahar* Africa or south* Africa or west* Africa or east* Africa).mp.  (Angola or Benin or Botswana or Burkina Faso or Burkina Fasso or Burundi or Cameroon or Cameroons or Cameron or Camerons or Cape Verde or Cabo Verde or Central African Republic or Chad or Comoros or Comoro Islands or Comores or Mayotte or Congo or Zaire or Cote d'Ivoire or Ivory Coast or Djibouti or French Somaliland or Eritrea or Estonia or Ethiopia or Gabon or Gabonese Republic or Gambia or Ghana or Kenya or Lesotho or Basutoland or Liberia or Madagascar or Malawi or Nyasaland or Mauritania or Mauritius or Mozambique or Namibia or Niger or Nigeria or Rwanda or Ruanda or Sao Tome or Senegal or Serbia or Seychelles or Sierra Leone or Somalia or South Africa or Sudan or South Sudan or Swaziland or Tanzania or Togo or Uganda or Zambia or Zimbabwe or Rhodesia).mp. |
| Intervention | Continuous Positive Airway Pressure/ or respiration, artificial/ or positive-pressure respiration  (bubble continuous positive airway pressure or bubble CPAP or BCPAP).mp.  (Pumani or Dolphin CPAP or Fisher Paykel or IntelliPAP or Besmed or WaterPAP or Babi-Plus).mp. |
