# Supplementary material for: Barriers and facilitators to implementing bubble CPAP to improve neonatal health in sub-Saharan Africa: a systematic review
Source: Public Health Rev. 2020 Apr 28;41:6. doi: 10.1186/s40985-020-00124-7 (PMC7189679; doi:10.1186/s40985-020-00124-7)
Supplement: Supplementary file 2 — Additional file 2:. Quality assessment tables. [file 40985_2020_124_MOESM2_ESM.docx]

**Additional file 2: Quality assessment tables**

| **Case series** | **Abdulkadir et al 2013** | **Abdulkadir et al 2015** | **Audu et al 2015** | **Brown et al 2013** | **McAdams et al 2016** | **Van Den Heuvel et al 2011** |
| --- | --- | --- | --- | --- | --- | --- |
| 1. Was the study question or objective clearly stated? | Yes | No | Yes | Yes | Yes | Yes |
| 2. Was the study population clearly and fully described, including a case definition? | Yes | Yes | Yes | No | No | Yes |
| 3. Were the cases consecutive? | N/A | Yes | Yes | No | Yes | CD |
| 4. Were the subjects comparable? | N/A | CD | CD | No | CD | CD |
| 5. Was the intervention clearly described? | No | Yes | Yes | Yes | Yes | Yes |
| 6. Were the outcome measures clearly defined, valid, reliable, and implemented consistently across all study participants? | No | Yes | No | Yes | Yes | CD |
| 7. Was the length of follow-up adequate? | No | Yes | No | CD | Yes | Yes |
| 8. Were the statistical methods well-described? | N/A | No | Yes | N/A | Yes | No |
| 9. Were the results well-described? | Yes | Yes | Yes | Yes | Yes | Yes |
| Rating | Poor | Fair | Fair | Poor | Fair | Fair |

N/A – not applicable
CD – cannot determine
NR –not reported

| **Observational cohort / cross-sectional studies** | **Gondwe, Gombachika and Majamanda 2017** | **Nabwera et al 2019** | **Nahimana et al 2015** | **Okonkwo and Okolo 2016** |
| --- | --- | --- | --- | --- |
| 1. Was the research question or objective in this paper clearly stated? | Yes | Yes | Yes | Yes |
| 2. Was the study population clearly specified and defined? | Yes | Yes | Yes | Yes |
| 3. Was the participation rate of eligible persons at least 50%? | CD | Yes | Yes | Yes |
| 4. Were all the subjects selected or recruited from the same or similar populations (including the same time period)? Were inclusion and exclusion criteria for being in the study prespecified and applied uniformly to all participants? | Yes | Yes | Yes | Yes |
| 5. Was a sample size justification, power description, or variance and effect estimates provided? | No | Yes | No | No |
| 6. For the analyses in this paper, were the exposure(s) of interest measured prior to the outcome(s) being measured? | Yes | Yes | Yes | No |
| 7. Was the timeframe sufficient so that one could reasonably expect to see an association between exposure and outcome if it existed? | CD | CD | Yes | No |
| 8. For exposures that can vary in amount or level, did the study examine different levels of the exposure as related to the outcome (e.g., categories of exposure, or exposure measured as continuous variable)? | No | N/A | No | N/A |
| 9. Were the exposure measures (independent variables) clearly defined, valid, reliable, and implemented consistently across all study participants? | N/A | N/A | Yes | N/A |
| 10. Was the exposure(s) assessed more than once over time? | N/A | N/A | CD | N/A |
| 11. Were the outcome measures (dependent variables) clearly defined, valid, reliable, and implemented consistently across all study participants? | N/A | Yes | Yes | N/A |
| 12. Were the outcome assessors blinded to the exposure status of participants? | N/A | No | No | N/A |
| 13. Was loss to follow-up after baseline 20% or less? | N/A | N/A | Yes | N/A |
| 14. Were key potential confounding variables measured and adjusted statistically for their impact on the relationship between exposure(s) and outcome(s)? | N/A | N/A | No | No |
| Rating | Poor | Fair | Fair | Poor |

| **Before-After (Pre-Post) Studies With No Control Group** | **Fulton 2013** | **Myhre et al 2016** | **Ntigurirwa et al 2017** |
| --- | --- | --- | --- |
| 1. Was the study question or objective clearly stated? | No | Yes | Yes |
| 2. Were eligibility/selection criteria for the study population prespecified and clearly described? | Yes | Yes | No |
| 3. Were the participants in the study representative of those who would be eligible for the test/service/intervention in the general or clinical population of interest? | CD | Yes | CD |
| 4. Were all eligible participants that met the prespecified entry criteria enrolled? | Yes | Yes | CD |
| 5. Was the sample size sufficiently large to provide confidence in the findings? | No | CD | CD |
| 6. Was the test/service/intervention clearly described and delivered consistently across the study population? | No | Yes | No |
| 7. Were the outcome measures prespecified, clearly defined, valid, reliable, and assessed consistently across all study participants? | Yes | Yes | No |
| 8. Were the people assessing the outcomes blinded to the participants' exposures/interventions? | CD | No | No |
| 9. Was the loss to follow-up after baseline 20% or less? Were those lost to follow-up accounted for in the analysis? | N/A | N/A | NR |
| 10. Did the statistical methods examine changes in outcome measures from before to after the intervention? Were statistical tests done that provided p values for the pre-to-post changes? | No | Yes | No |
| 11. Were outcome measures of interest taken multiple times before the intervention and multiple times after the intervention (i.e., did they use an interrupted time-series design)? | No | Yes | Yes |
| 12. If the intervention was conducted at a group level (e.g., a whole hospital, a community, etc.) did the statistical analysis take into account the use of individual-level data to determine effects at the group level? | N/A | N/A | N/A |
| Rating | Poor | Fair | Poor |

| **Controlled intervention study** | **Amadi et al 2019** | **Crehan et al 2018** | **Kawaza et al 2014** | **Olayo et al 2019** |
| --- | --- | --- | --- | --- |
| 1. Was the study described as randomized, a randomized trial, a randomized clinical trial, or an RCT? | No | No | No | No |
| 2. Was the method of randomization adequate (i.e., use of randomly generated assignment)? | N/A | N/A | N/A | N/A |
| 3. Was the treatment allocation concealed (so that assignments could not be predicted)? | N/A | N/A | N/A | N/A |
| 4. Were study participants and providers blinded to treatment group assignment? | No | No | No | No |
| 5. Were the people assessing the outcomes blinded to the participants' group assignments? | No | No | No | No |
| 6. Were the groups similar at baseline on important characteristics that could affect outcomes (e.g., demographics, risk factors, co-morbid conditions)? | No | No | Yes | Yes |
| 7. Was the overall drop-out rate from the study at endpoint 20% or lower of the number allocated to treatment? | NR | Yes | Yes | NR |
| 8. Was the differential drop-out rate (between treatment groups) at endpoint 15 percentage points or lower? | NR | Yes | Yes | N/A |
| 9. Was there high adherence to the intervention protocols for each treatment group? | NR | Yes | Yes | CD |
| 10. Were other interventions avoided or similar in the groups (e.g., similar background treatments)? | NR | NR | NR | NR |
| 11. Were outcomes assessed using valid and reliable measures, implemented consistently across all study participants? | CD | Yes | Yes | No |
| 12. Did the authors report that the sample size was sufficiently large to be able to detect a difference in the main outcome between groups with at least 80% power? | No | No | Yes | Yes |
| 13. Were outcomes reported or subgroups analyzed prespecified (i.e., identified before analyses were conducted)? | No | No | No | No |
| 14. Were all randomized participants analyzed in the group to which they were originally assigned, i.e., did they use an intention-to-treat analysis? | N/A | N/A | Yes | N/A |
| Rating | Poor | Poor | Fair | Poor |
