# Supplementary material for: Barriers and facilitators to implementing bubble CPAP to improve neonatal health in sub-Saharan Africa: a systematic review
Source: Public Health Rev. 2020 Apr 28;41:6. doi: 10.1186/s40985-020-00124-7 (PMC7189679; doi:10.1186/s40985-020-00124-7)
Supplement: Supplementary file 3 — Additional file 3:. Primary objective. [file 40985_2020_124_MOESM3_ESM.docx]

**Additional file 3: Primary objective**

**Barriers and facilitators by study**

| **Reference** | **Facilitators of implementation** | **Barriers of implementation** |
| --- | --- | --- |
| Abdulkadir et al 2013 | - Ease of use: Simple - Affordable: Inexpensive - Supplies and equipment: Appropriate snug-fitting nasal prongs to prevent complications | - Weaning: Deterioration of condition after initial weaning - Monitoring: Importance of close monitoring to be aware if desaturating |
| Abdulkadir et al 2015 | - Ease of use: Simple (in comparison to mechanical ventilator care) | - Initiation: Meticulous attention needed for application, positioning and tightness of chin strap - Monitoring: CPAP belly syndrome requires treatment by intermittent orogastric intubation to decompress stomach - Limited efficacy: Efficacy may be limited to mild to moderate respiratory distress (2 of the 3 who died had severe RDS and high Downes’ score) |
| Amadi et al 2019 | - Affordable -The politeCPAP costs around US$2000 whereas standard commercial CPAP brands in Nigeria range from US$5000 to US$18,000 - Device characteristics: a temperature-controlled gas circuit to ensure that newborns do not experience significant thermal instability | - Supplies and equipment: five technical call-outs for system breakdown during the initial 3-month clinical trial |
| Audu et al 2015, Audu et al 2013 | - Ease of use: Simple and easy to use - Affordable: Inexpensive – average cost of disposable components of the device was N2,000 ($12), average cost of providing consumables (corrugated tubes, nasal prongs, head caps) was N8,000 ($48) per patient - Supplies and equipment: Locally adapted device used materials that were readily available and did not need to be sourced separately - Supplies and equipment: Soft nasal prongs – does not cause ischaemic damage to nasal septum - Monitoring: Clinical assessment on admission and at 1, 6 and 12 hours after commencement on CPAP - Monitoring: Usefulness of pulse oximetry as a monitoring tool as the rapid improvement in oxygenation preceded the significant reduction in respiratory rate | - Limited efficacy: Efficacy may be limited to mild to moderate respiratory distress (majority of newborns who did not respond to CPAP were extremely low birthweight or had cyanotic congenital heart disease or recurrent apnoea and severe hypoxic ischaemic encephalopathy) |
| Brown et al 2013 | - Affordable: cost of good to fabricate a single device at low production volume was approximately $350 USD | - Monitoring: the device does not heat or humidify the pressurized air delivered to the infant and needs regular use of nasal saline drops to prevent mucosal drying - Supplies and equipment: Accessing ancillary equipment and disposable supplies may be a barrier. Cost of disposable nasal prongs likely a barrier to scale up in low-resource settings - Parental perceptions: parents are reluctant to allow their children to receive any oxygen therapy because need for oxygen associated with a poor outcome |
| Crehan et al 2018 | - Initiation/ decision-making: TRY clinical algorithm (Tone: good, Respiratory distress, Yes, heart rate above 100 breaths/min) found to have high inter-rater reliability between nurses and visiting paediatrician - Initiation/decision-making: TRY algorithm attached to bCPAP machines - Training: Weekly interactive teaching sessions including practicing use in theoretical case vignettes and real-time supervision on one real infant was conducted by visiting paediatrician before and during study period | - Staffing: Discussed that CPAP may overstretch the unit’s capacity to deliver good care if basic neonatal nursing needs are not being met |
| Fulton and Lavalette 2014 | - Training: To improve motivation, nurses were taken to a particularly effective neonatal unit as an “experience sharing trip” - Staffing: Investing in nurses who are more constant in the ward (vs doctors) | - Staffing: High turnover of junior doctors through the unit was detrimental to sustainability - Staffing: Lack of staff and poor resources led to existing substantial workloads, lack of motivation and poor basic care - Training: Nursing staff did not use learnings from regular teaching and training sessions to a severe lack in motivation and accountability - Medical team cohesiveness: Communication barrier between local doctors who had medical meetings and ward rounds in English and nurses who did not understand |
| Gondwe, Gombachika and Majamanda al 2017 | - Not given specifically for neonates (Chatinkha nursery). Results from combination of Chatinkha and paediatric nursery include information to help them understand infant’s condition, when nurses welcomed parental participation, psychological support from health care workers and support from close family members so information should be shared with them too | - Initiation and information sharing: Because in Chatinkha Nursery, caregivers stay in a different ward and visit neonates every three hours, information was usually received after commencing and participants noted that information was only provided upon asking. Caregivers who found their infants already started on bCPAP were more stressed - Mother-infant interaction: caregivers reported that bubble CPAP device further complicated their interaction with newborn (mothers afraid to hold babies, unable to see their infant’s faces and skin-to-skin contact is interrupted), which was already stressed with Chatinkha visit policy |
| Kawaza et al 2014, Chen et al 2014 | - Highly cost effective: Average cost per patient was US $29.29 (SD 26.62) for a patient on nasal oxygen and $57.78 (SD 40.92) but incremental effectiveness associated with BCPAP was 6.78 life years in comparison with nasal oxygen. Incremental cost-effectiveness ratio for bCPAP relative to nasal oxygen was $4.20 (95% CI 2.29-16.67) per life year gained. - Low maintenance: Therapeutic flow and pressure met performance standards reliably without preventative maintenance and no device failures occurred | - Initiation: Optimal time to initiate treatment with bubble CPAP unclear, the average delay in receiving bubble CPAP was 3.1 days and the neonates (n=9) who transitioned from nasal oxygen to bubble CPAP had twice as long average hospital stays (22 days vs 14 bubble CPAP) and twice as long on treatment (14 days vs 7 days bubble CPAP) - Monitoring: Sterile nasal saline drops needed to be administered every four hours to reduce mucosal drying - Supplies and equipment: 40% of study oxygen concentrators failed when circuit boards were damaged by line voltage spikes |
| McAdams et al 2015 | - Training: short training period where NICU physician scored 38 NICU subjects simultaneously with nurses and video with a infant with respiratory distress shown and scored by physician and nurses - Training: Training for respiratory severity score (RSS) completed over the course of one week through a series of educational workshops while on the job - Monitoring: Silverman-Andersen respiratory severity score (RSS) useful for monitoring a neonate’s respiratory status and had 0.73 correlation of scoring between doctor and nurse after short training | - Staffing: Nurse to neonate ratios is important to understand and need to further understand whether nurse and physician time allocation to sick infant on bubble CPAP detracts from care of other infants in the NICU - Weaning: Weaning parameters must be established for optimal respiratory management, especially in a resource-limited setting |
| Myhre et al 2016 | - Training: Training required for introduction of bubble CPAP likely enabled nurses to improve their general neonatal nursing skills as evidenced by lower referral rate of preterm infants with RDS in the bubble CPAP period (4% vs 17%, *p*= 0.037) | - N/A |
| Nabwera et al 2019 | - Staffing: good leadership by health facility management that prioritized neonatal care - Training and mentorship: Provider to provider mentorship to new staff by CPAP “champions” - Caregivers: peer support from caregivers with positive experiences with bubble CPAP use on their own newborns | - Training: inadequate training of healthcare providers on the use of bubble CPAP - Staffing: Healthcare provider strikes, high staff turnover, ongoing staff shortages - Supplies and equipment: poor equipment maintenance once donors withdraw support, inadequate infrastructure lowers staff morale and motivation to use bubble CPAP |
| Nahimana et al 2015 | - Training and mentorship: Training was supplemented by ongoing clinical mentorship and intermittent refresher trainings led by Partners in Health (PIH) and local Ministry of Health (MOH) bubble CPAP champions | - Staffing: High turnover of nurses and doctors - Staffing: No full time pediatric specialist on staff - Initiation: Ongoing gaps in correct identification of early and mild signs of distress and initiation of eligible infants revealed as on 52% (n=43/83) received bubble CPAP. Of these, 39% (n=9) died |
| Ntigurirwa et al 2017 | - Staffing: Introduction of some longer-term clinicians to the program allowed for the introduction of CPAP, development of new medical records and assisting with the audits as well as with reinforcing the training - Diffusion of innovation: Demand for CPAP rapidly increased once introduced into the country | - Supplies and infrastructure: Different machines being introduced each required its own set of guidelines, training, different circuits and maintenance - Staffing: the very small number of nurses working at each neonatal unit limited their ability to provide care - Training: small number of nurses limited nursing staff availability for training and frequent staff turnover (both nurses and doctors) necessitated repeated training of new staff and severely impeded institutional memory - Training: New nurses received minimal training for neonatal care as a part of their general nurses training - Motivation: lack of motivation of local medical and nursing leadership and staff, exacerbated by frequent moves to other departments |
| Okonkwo and Okolo 2016 | - Affordable: improvised Nigerian device cost less than $2 to assemble a unit. It is one of the cheapest improvised devices | - Training: Improvised CPAP training undertaken by 51% (n=121) of respondents and 44% pf respondents not trained - Equipment and supplies: Private facilities largely used patented bubble CPAP systems while only a third of public tertiary facilities had patented bubble CPAP systems, which is likely due to high cost of the patented machines ($6000). Some centres used both improvised and patented CPAP machines |
| Olayo et al 2019 | - Training: Effective training-the-trainer model with a two day workshop (didactic and simulation sessions on day one and taught how to train others to use bubble CPAP on day two). Study found no statistically significant differences between first- and second-generation healthcare providers’ skills (90%, 95% CI 87–93 vs 89%, 95% CI 86–92) or knowledge scores (91%, 95% CI 88–93 vs 90%, 95% CI 88–93) when second-generation was assessed six months after training the first-generation | - Staffing: high turnover rates of medical staff, frequent transfer to other government health facilities, scheduled leave and clinical responsibilities |
| van den Heuvel et al 2011 | - Training: A neonatal intensive care nurses from the Netherlands was recruited and provided multiple teaching sessions with a local paediatrician. Nurse continued to work intensively with local neonatal nurses for 6 weeks | - Staffing: shortage of staff as only two nurses available during the day and night with an average of 30 neonates and 10 admissions a day. Due to the short staffing, nurses were reluctant to use CPAP particularly at night when medical students, student nurses, residents and other medical staff were not present - Initiation: Nurses reluctant to put infant on CPAP without first consulting a doctor and use of CPAP declined after the initial training period - Initiation: Six neonates who did not fulfill the inclusion criteria received CPAP - Supplies and equipment: 31% (n=12) died because they did not receive CPAP as the system was occupied. |
