# Supplementary material for: Barriers and facilitators to implementing bubble CPAP to improve neonatal health in sub-Saharan Africa: a systematic review
Source: Public Health Rev. 2020 Apr 28;41:6. doi: 10.1186/s40985-020-00124-7 (PMC7189679; doi:10.1186/s40985-020-00124-7)
Supplement: Supplementary file 4 — Additional file 4:. Secondary objectives. [file 40985_2020_124_MOESM4_ESM.docx]

**Additional file 4: Secondary objectives**

**Different types of bubble CPAP being use and facility levels they are currently being implemented in Sub-Saharan Africa (secondary objective 1)**

| **Bubble CPAP device** | **Facility level** | | |
| --- | --- | --- | --- |
|  | **Tertiary level hospital** (n= 11 studies) | **Secondary level rural referral or district hospital**  (n=4 studies) | **Multi-site including both tertiary and district hospitals** (n=2 studies) |
| **Improvised water bottle system**  (n=6 studies) | Abdulkadir et al 2015, Audu et al 2015, Fulton and Lavalette 2014, van den Heuvel et al 2011 | McAdams et al 2015, Myhre et al 2016 | N/A |
| **Low-lcost standalone system**  (n=5 studies) | Amadi et al 2019, Brown et al 2013, Gondwe, Gombachika and Majamanda al 2017, Kawaza et al 2014/Chen et al 2014 | Crehan et al 2018 | N/A |
| **Commercial bubble CPAP system** (n=2) | Olayo et al 2019 | N/A | Ntigurirwa et al 2017 |
| **Bubble CPAP system not described or combination**  (n=4 studies) | Abdulkadir et al 2013, Nabwera et al 2019 | Nahimana et al 2015 | Okonkwo and Okolo 2016 |

**Barrier and facilitators by bubble CPAP device type and facility level (secondary objective 2)**

| **Bubble CPAP device** | **Facility level** | | |
| --- | --- | --- | --- |
|  | **Tertiary level hospital** (n= 11 studies) | **Secondary level rural referral or district hospital**  (n=4 studies) | **Multi-site including both tertiary and district hospitals** (n=2 studies) |
| **Improvised water bottle system**  (n=6 studies) | Facilitators   - **Simple** to use (Abdulkadir et al 2015, Audu et al 2015 - **Inexpensive**, ~$48 USD, in comparison to Pumani model (Audu et al 2015) - **Locally available materials** can be used (Audu et al 2015) - **Use of soft nasal prongs** (Audu et al 2015 - **Appropriate and regular monitoring** (Audu et al 2015) - **Use of pulse oximetry for monitoring** (Audu et al 2015) - **Clinical mentorship and investing in nurses** (Fulton and Lavalette 2014)   Barriers   - **Efficacy may be limited to mild to moderate respiratory distress** and less effective with severe cases (Abdulkadir et al 2015, Audu et al 2015) - **Complications such as CPAP belly syndrome** require regular monitoring and actions to prevent (Abdulkadir et al 2015) - **Overtightening the chin strap can lead to facial swelling** (Abdulkadir et al 2015) - **High turnover of clinical staff** (Fulton and Lavalette 2014) - **Understaffed neonatal units** limit capacity for care (Fulton and Lavalette 2014, van den Heuvel et al 2011) - **Lack of motivation and accountability** (Fulton and Lavalette 2014) - **Communication barriers** between local doctors who worked in English and nurses who did not understand (Fulton and Lavalette 2014) - **Reluctance of nurses to initiate** while short staffed at night, and without first consulting a doctor (van den Heuvel et al 2011) - **Lack of available CPAP systems** (van den Heuvel et al 2011) | Facilitators   - **Short training period** appeared to be successful for implementation by nurses and doctors (McAdams et al 2015) - **Monitoring respiratory distress with respiratory severity score** (McAdams et al 2015)   Barriers   - **Understaffed neonatal units** limit capacity for care (McAdams et al 2015) - **Knowing when to wean**, especially when resources are limited (McAdams et al 2015) | N/A |
| **Low-cost standalone system**  (n=4 studies) | Facilitators   - **Low maintenance** (Kawaza et al 2014) - **Inexpensive**, ~$350 /$2000 USD in comparison to commercial models that range from $5000-18,000 USD (Brown et al 2013, Amadi et al 2019) and **highly cost effective** (Chen et al 2104) - **A temperature-controlled gas circuit** may reduce risk of hypothermia especially in low birthweight babies (Amadi et al 2019)   Barriers   - **The device does not heat or humidify** the pressurized air delivered to the infant and needs regular use of nasal saline drops to prevent mucosal drying (Brown et al 2013, Kawaza et al 2014) - **Cost of disposable nasal prongs** (Brown et al 2013) - **Oxygen concentrators not always available** (Kawaza et al 2014) - **Local beliefs that the oxygen led to poor outcomes** (Brown et al 2013) - **Information about bubble CPAP was provided to caregivers sporadically**, often upon request and after commencement of bCPAP. Caregivers who found their infants already started on bcPAP were more stressed than those informed beforehand (Gondwe, Gombachika and Majamanda 2017) - **Bubble CPAP may complicate mother-infant interaction** as mothers were afraid to hold babies, unable to see their infant’s faces and interrupted skin-to-skin contact (Gondwe, Gombachika and Majamanda 2017) | Facilitators   - **Decision-making aided by clinical algorithm** that is clearly posted by machine (Crehan et al 2018) - **Regular and interactive training** (Crehan et al 2018)   Barriers   - **Understaffed neonatal units** limit capacity for care (Crehan et al 2018) | N/A |
| **Commercial bubble CPAP system**  (n=2) | Facilitators   - **Training-the-trainers clinical mentorship** with training on how to train others on bubble CPAP use (Olayo et al 2019) - **Interactive training** (Olayo et al 2019)   Barriers   - **High turnover rates of medical staff** and frequent transfer to other government health facilities, scheduled leave and clinical responsibilities (Olayo et al 2019) | N/A | Facilitators   - **Once introduced into the country, demand for CPAP rapidly increased** (Ntiguriwa et al 2017) - **Introduction of some longer term clinicians** helped reinforce the training and supported the program (Ntiguriwa et al 2017)   Barriers   - **High turnover of clinical staff** (Ntigurirwa et al 2017) - **Understaffed neonatal units** limit capacity for care (Ntigurirwa et al 2017) - **New nurses received minimal training** for neonatal care as a part of their general nurses training (Ntigurirwa et al 2017) - **Lack of motivation** of local medical and nursing leadership exacerbated by frequent departmental moves (Ntigurirwa et al 2017) |
| **Bubble CPAP system not described or combination**  (n=3 studies) | Facilitators   - **Simple and inexpensive** (Abdulkadir et al 2013) - **Having appropriate snug-fitting nasal prongs** (Abdulkadir et al 2013) - **Good hospital management leadership** that prioritizes neonatal care (Nabwera et al 2019) - **Mentorship by bubble CPAP “champions”** (Nabwera et al 2019) - **Peer support** by caregivers who had positive experiences with bubble CPAP with their newborns (Nabwera et al 2019)   Barriers   - **Inadequate training** (Nabwera et al 2019) - **High staff turnover and ongoing staff shortages** (Nabwera et al 2019) - **A need to monitor closely after weaning** to ensure infant is not desaturating (Abdulkadir et al 2013) - **Poor equipment maintenance** once donors withdraw support (Nabwera et al 2019) | Facilitators   - **Ongoing clinical mentorships and intermittent refresher trainings** (Nahimana et al 2015) - **Combination of external consultant trainers with local clinicians as trainers** (Nahimana et al 2015)   Barriers   - **High turnover of nurses and doctors** (Nahimana et al 2015) - **No full time pediatric specialist on staff** (Nahimana et al 2015) - **Ongoing gaps in correct identification of early and mild signs of distress** and initiation of eligible infants (Nahimana et al 2015) | Facilitators   - **Affordable** – improvised Nigerian device cost less than $2 to assemble a unit. It is one of the cheapest improvised devices (Okonkwo and Okolo 2016)   Barriers   - **Gaps in training** as many nurses and doctors are untrained in bubble CPAP (Okonkwo and Okolo 2016) - **Private facilities** largely used patented bubble CPAP systems, which was less available in public facilities (Okonkwo and Okolo 2016) - **Some centres used both improvised and patented CPAP machines** (Okonkwo and Okolo 2016) |

**Efficacy and safety by study (secondary objective 3)**

| **Reference** | **Effectiveness** | **Safety** | **Availability of mechanical ventilation** |
| --- | --- | --- | --- |
| Abdulkadir et al 2013 | - Successfully weaned off after 60 hours total on bubble cpap - A repeat chest radiograph showed aeration of the lungs in comparison to an earlier one featuring a reticulogranular appearance | - No complications (such as of pneumothorax, nasal septal erosion or necrosis, and gastric distention) | - N/A (commented that NICU units often lack ventilator support in Nigeria and even when they do, inadequate skilled staffing to provide the care) |
| Abdulkadir et al 2015 | - 85.0% (n=17) successfully weaned off CPAP (10 of which subsequently died of other non-respiratory complications so overall survival rate was only 41%) - 15.0% (n=3) died (two of which may have been associated with CPAP failure, third had congenital pneumonia) | - 10% (n=2) had CPAP belly syndrome - 5% (n=1) had facial swelling from chin strap, which resolved spontaneously within 6 hrs of adjusting chin strap | - Mechanical ventilation not available (commented that the three neonates who failed CPAP had more severe disease and required higher respiratory support in form of mechanical ventilation, which was not available) |
| Amadi et al 2019 | - 95.5% (21 out of 22) babies on politeCPAP alive - 65.7% (23 out of 35) babies on improvised bubble CPAP system alive (significance testing between the two groups not completed) - For extremely low birthweight babies, all six babies on polite CPAP survived while all nine babies on improvised bubble CPAP system died | N/A | N/A |
| Audu et al 2015, Audu et al 2013 | - 79.2% (n=38) survived to discharge - 20.8% (n=10) mortality rate | N/A | - Mechanical ventilation not available (babies would otherwise have received supplemental intranasal oxygen) |
| Brown et al 2013 | - Survived to discharge - Initial oxygen saturation was 55%, increased to 93% within four hours of initiating bubble CPAP | - No evidence of mucosal drying or other complications observed | N/A |
| Crehan et al 2018 | - 56% (n=32) survival to discharge - 42% (n=24) mortality rate (prematurity and RDS 54%, severe birth asphyxia 33%, prematurity 8% and meconium aspiration 45) | - No significant complications (such as pneumothorax) | N/A |
| Fulton and Lavalette 2014 | N/A (states CPAP was effective but does not explain further) | N/A | N/A |
| Gondwe, Gombachika and Majamanda al 2017 | N/A | N/A | N/A |
| Kawaza et al 2014, Chen et al 2014 | - survival to discharge: 71.0% (44 out of 62) in the bCPAP group in comparison to 44.0% (11 out of 25) in the nasal oxygen group (p=0.018) - 27% absolute improvement in survival - 65.5% vs 15.4% among very low birth weight neonates - 64.6% vs 23.5% among neonates with RDS - 61.5% vs 0% among neonates with sepsis | - No clinical diagnoses of pneumothorax - 1.9% (n=1) of those initially started on bubble CPAP and 0% of those transitioned to bubble CPAP had mild facial irritation, <1 day duration - 13.2% (n=7) of those initially started on bubble CPAP and 22.2% (n=2) of those transitioned to bubble CPAP had mild nasal irritation, <3 day duration - 11.3% (n=6) of those initially started on bubble CPAP and 22.2% (n=2) of those transitioned to bubble CPAP had mild epistaxis | - Mechanical ventilation not available (alternative treatment was nasal oxygen) |
| McAdams et al 2015 | - 52.4% (11 out of 21 infants) survived to discharge - RDS infants had a 62.5% survival rate (10 out of 16) - Birth asphyxia neonates had a 40% (2 out of 5) survival rate. - Definitive cause of death in the 9 who died is unknown since there were no autopsies | - No major complications - 14% (n=3) neonates diagnosed with nasal irritation after bubble CPAP was started | - Mechanical ventilation not available (alternative treatment was nasal oxygen) |
| Myhre et al 2016 | - Survival to discharge was significantly better during the bCPAP period (85% vs 61%, p =0.007; OR 3.33, 95%CI: 1.42-10.0) - 11% (7 out of 72) died during the bCPAP period compared to 22% (10 out of 46) in the period before, but the difference was not significant | N/A | N/A |
| Nabwera et al 2019 | - 51% (20 of 39) died - 49% (19 of 39) survived to discharge | - 21% (8/39) were diagnosed with necrotising enterocolitis - 8% (3/39) were reported to have nasal lesions. - No other complications were reported. | - Available in 37% (7 of 19) hospitals surveyed |
| Nahimana et al 2015 | - 41.9% (n=18) recovered/discharged - 48.8% (n=21) died - 6.9% (n=3) transferred for higher level care | - No complications (such as skin injury, pneumothorax or abdominal distention) | - Mechanical ventilation not available on site. - Infants who needed intensive neonatal care, including mechanical ventilators, were referred to tertiary hospitals |
| Ntigurirwa et al 2017 | - N/A (neonatal mortality reduced from 24 to 22% at university hospitals and from 10% to 8% at district hospitals for combined neonatal package, results for bubble CPAP not separated) | - 13% transient nasal or facial trauma attributed to excessive pressure applied by the cannule in the nose or by the face mask to the nasal bridge - 2% significant abdominal distension - No reports of pneumothorax - Technical problems minor and occurred in only 2% of infants | N/A |
| Okonkwo and Okolo 2016 | N/A | N/A | - Least available mode of respiratory support in Nigerian health facilities - Mechanical ventilation reported by 13.5% (n=32) respondents (but unclear how many facilities this represents) |
| Olayo et al 2019 | N/A (neonates mixed with pediatric patients though median age was 1 day) | N/A | N/A |
| van den Heuvel et al 2011 | - Three out of the five (60%) who met the inclusion criteria survived | - No major complications - A little nasal soreness or abrasion - Hypoxia caused by nasal obstruction was a problem and necessitated frequent suction | - Mechanical ventilation not available (commented that one died on CPAP because no further respiratory support was available) |

**Efficacy by bubble CPAP device type and facility level (secondary objective 3)**

| **SURVIVAL TO DISCHARGE RATES (excludes case studies; n=8, 57.1% of studies discussed survival)** | | | |
| --- | --- | --- | --- |
| **Bubble CPAP device** | **Facility level** | | |
|  | **Tertiary level hospital** (n= 5 studies) | **Secondary level rural referral or district hospital**  (n=4 studies) | **Multi-site including both tertiary and district hospitals** (n=0 studies) |
| **Improvised water bottle system**  (n=5 studies) | - 85% (Abdulkadir et al 2015) - 79.2% (Audu et al 2015) - 60% (Van den Heuvel et al 2011) | - 52.4% (McAdams et al 2015) - 85% after bCPAP implemented vs 61% before (p=0.007) (Myhre et al 2016) | N/A |
| **Low-cost standalone system**  (n=3 studies) | - 71.0% in bCPAP group vs 44.0% in nasal oxygen (p=0.018) (Kawaza et al 2014) | - 56.1% (Crehan et al 2018) | N/A |
| **Commercial bubble CPAP system** (n=0) | N/A | N/A | N/A |
| **Bubble CPAP system not described or combination**  (n=1 studies) | - 49% (Nabwera et al 2019) | - 41.9% (Nahimana et al 2015) | N/A |

**Safety by bubble CPAP device type and facility level (secondary objective 3)**

| **COMPLICATIONS REPORTED (n=9, 64.3% of studies discussed complications)** | | | |
| --- | --- | --- | --- |
| **Bubble CPAP device** | **Facility level** | | |
|  | **Tertiary level hospital** (n= 5 studies) | **Secondary level rural referral or district hospital**  (n= 3 studies) | **Multi-site including both tertiary and district hospitals** (n= 1 studies) |
| **Improvised water bottle system**  (n= 3 studies) | - 10% (n=2) had CPAP belly syndrome and 5% (n=1) had facial swelling from chin strap, which resolved spontaneously within 6 hrs of adjusting chin strap (Abdulkadir et al 2015) - No major complications; a little nasal soreness or abrasion; hypoxia caused by nasal obstruction was a problem and necessitated frequent suction (van den Heuvel et al 2011) | - No major complications; 14% (n=3) neonates diagnosed with nasal irritation after bubble CPAP was started (McAdams et al 2015) | N/A |
| **Low-cost standalone system**  (n=3 studies) | - No evidence of mucosal drying or other complications observed (Brown et al 2013) - No clinical diagnoses of pneumothorax; 1.9% (n=1) of those initially started on bubble CPAP and 0% of those transitioned to bubble CPAP had mild facial irritation, <1 day duration; 13.2% (n=7) of those initially started on bubble CPAP and 22.2% (n=2) of those transitioned to bubble CPAP had mild nasal irritation, <3 day duration; 11.3% (n=6) of those initially started on bubble CPAP and 22.2% (n=2) of those transitioned to bubble CPAP had mild epistaxis (Kawaza et al 2014) | - No significant complications such as pneumothorax (Crehan et al 2018) | N/A |
| **Commercial bubble CPAP system**  (n= 1) | N/A | N/A | - No reports of pneumothorax; 13% transient nasal or facial trauma attributed to excessive pressure applied by the cannule in the nose or by the face mask to the nasal bridge; 2% significant abdominal distension; technical problems minor and occurred in only 2% of infants (Ntigurirwa et al 2017) |
| **Bubble CPAP system not described or combination**  (n= 2 studies) | - No complications such as of pneumothorax, nasal septal erosion or necrosis, and gastric distention (Abdulkadir et al 2013) - 8% (3/39) were reported to have nasal lesions (Nabwera et al 2019) - 21% (8/39) were diagnosed with necrotising enterocolitis (Nabwera et al 2019) | - No complications such as skin injury, pneumothorax or abdominal distention (Nahimana et al 2015) | N/A |
